# An Endoscopic Illusion!

**DOI:** 10.14309/crj.0000000000001278

**Published:** 2024-03-16

**Authors:** Victoria Sandoval, Vibhu Chittajallu, Fady G. Haddad

**Affiliations:** 1 Department of Internal Medicine, Case Western Reserve University, University Hospitals Cleveland Medical Center, Cleveland, OH; 2 Department of Gastroenterology and Hepatology, Case Western Reserve University, University Hospitals Cleveland Medical Center, Cleveland, OH

**Keywords:** peptic ulcer disease, double pylorus, gastroduodenal fistula, endoscopy

## CASE REPORT

**Figure 1:**
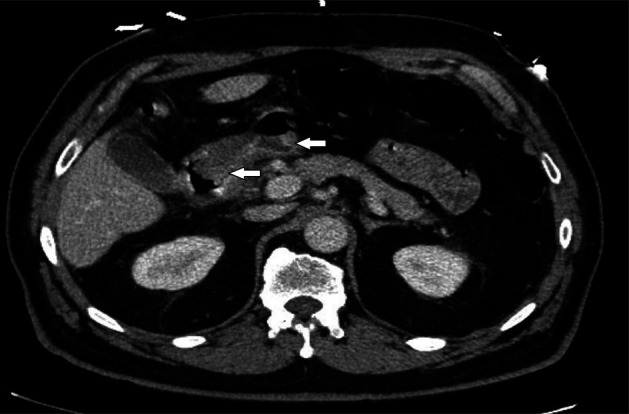
Abdominal CT scan showing inflammatory changes of the gastric antrum and proximal duodenum.

**Figure 2:**
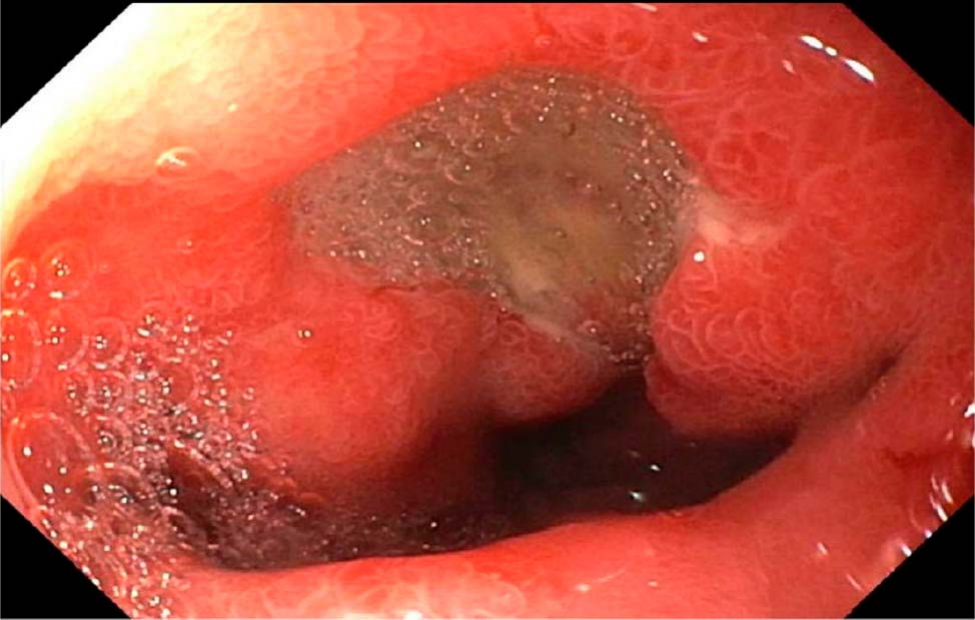
Upper endoscopy showing a 7 mm cratered duodenal bulb ulcer.

**Figure 3:**
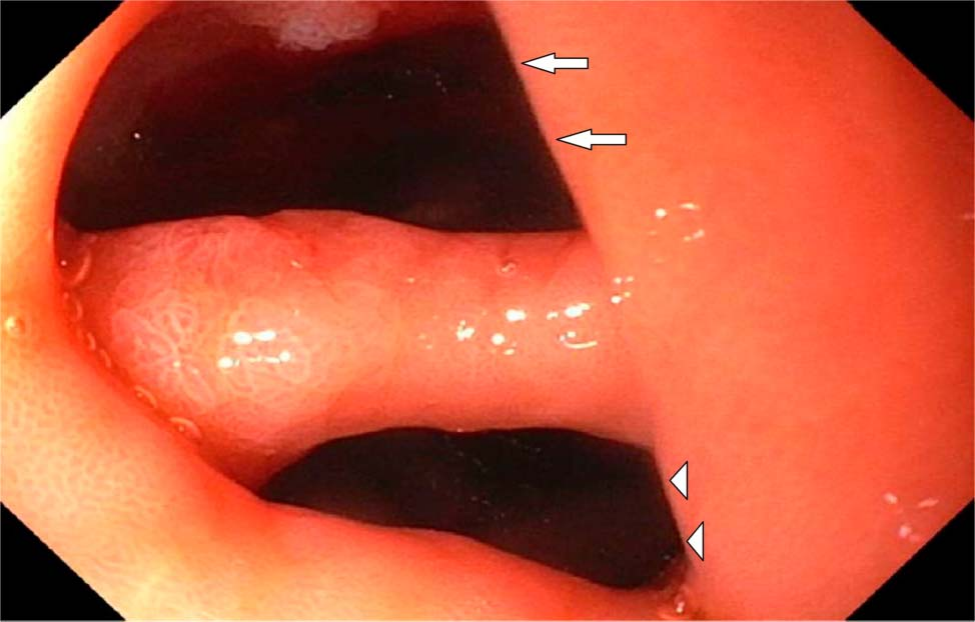
Upper endoscopy showing an accessory pyloric duct (arrows) connecting the gastric antrum to the proximal duodenum that is separated from the true pyloric duct (arrowheads) by a bridging tissue.

A 74 year-old man with hypertension and benign prostatic hyperplasia presented with few week history of epigastric pain and vomiting. He denied any history of *Helicobacter pylori* infection, chronic gastritis, peptic ulcer disease, or endoscopic procedures in the past. Home medications included amlodipine and tamsulosin. He denied using any alcohol, illicit drugs, nonsteroidal anti-inflammatory drugs, corticosteroids, bisphosphonates, iron or potassium supplements, or chronic antibiotics. Physical examination revealed epigastric tenderness. An abdominal computed tomography scan showed wall thickening involving the gastric antrum and proximal duodenum with surrounding fat stranding (Figure 1, arrows). Esophagogastroduodenoscopy revealed gastric erythema, a 7 mm cratered duodenal bulb ulcer, and a gastroduodenal fistula that connected the gastric antrum to the proximal duodenum consistent with a double pylorus (Figure 2). The fistula created an accessory pyloric duct that was separated from the true pyloric duct by a bridging tissue (Figure 3, arrows, arrowheads). Routine staining of gastric biopsies identified *H. pylori* organisms.

He received high-dose proton pump inhibitors and *H. pylori* eradication and was advised to avoid nonsteroidal anti-inflammatory drugs. Symptoms significantly improved after few days of treatment.

Double pylorus is a rare endoscopic finding estimated to be seen in <0.4% of esophagogastroduodenoscopies. Its true incidence in the population is unknown since it is usually noted as an incidental finding. It is the presence of a double connection between the antrum and the proximal duodenum through a gastroduodenal fistula.^1^ It can be congenital or acquired.^1,2^ Congenital cases are associated with gastric duplication, heterotrophic pancreatic tissue, and pancreas divisum.^2^ Acquired cases are secondary to systemic diseases, drugs, or *H. pylori* infection.^1,2^ Double pylorus can manifest as gastrointestinal bleeding or abdominal pain.^2^ Treatment includes acid suppression through proton pump inhibitors or H2-receptor antagonists.^2^ Refractory cases require advanced endoscopic or surgical interventions.^2^

## DISCLOSURES

Author contributions: V. Sandoval wrote the manuscript and reviewed the literature. V. Chittajallu edited the manuscript. FG Haddad wrote and edited the manuscript and acquired the images. All authors approved the final draft submitted. FG Haddad is the article guarantor.

Financial disclosure: None to report.

Previous presentation: The case report was presented at the ACG Annual Scientific Meeting; October 24, 2022; Charlotte, North Carolina.

Informed patient consent was obtained for this case report.
